# Supplementary material for: Variation and Abundance of Resistant Starch in Selected Banana Cultivars in Uganda
Source: Foods. 2024 Sep 21;13(18):2998. doi: 10.3390/foods13182998 (PMC11431251; doi:10.3390/foods13182998)
Supplement: Supplementary file 1 [file foods-13-02998-s001.zip › foods-3122344-supplementary.pdf]

Table S1: Percentage transmittance across samples from 0-98 hours

Samples	0hr	24hr	48hr	72hr	98hr
ddH2O	100 ± 0^{A}	100 ± 0^{A}	100 ± 0^{A}	100 ± 0^{A}	100 ± 0^{A}
Sukali Ndiizi	89.331 ± 0.4^{B}	91.658 ± 0.6^{B}	92.151 ± 0.3^{B}	93.1465 ± 0.1^{B}	93.829 ± 0.5^{B}
Kivuvu	78.859 ± 0.8^{C}	74.849 ± 0.9^{C}	77.894 ± 0.3^{C}	77.785 ± 1.6^{D}	64.149 ± 0.9^{D}
Kayinja	76.15 ± 0.2^{C}	71.6419 ± 0.1^{D}	69.211 ± 0.5^{D}	63.9981 ± 0.1^{EF}	41.607 ± 0.2^{J}
Gonja	69.13 ± 4.1^{D}	54.639 ± 0.3^{IJ}	55.251 ± 0.5^{H}	56.952 ± 0.4^{H}	40.029 ± 0.6^{J}
Nakitembe	67.739 ± 0.5^{DE}	60.978 ± 0.4^{F}	66.147 ± 0.5^{E}	66.711 ± 0.9^{E}	51.39 ± 1.9^{H}
Kibuzi	67.303 ± 1.0^{DEF}	64.595 ± 0.9^{E}	65.969 ± 0.5^{E}	85.969 ± 0.7^{C}	93.7922 ± 0.1^{B}
G. Michel	65.389 ± 0.3^{EFG}	60.258 ± 0.6^{FG}	62.183 ± 0.5^{F}	61.249 ± 1.6^{FG}	57.969 ± 0.8^{FG}
Nfuuka	64.692 ± 0.8^{EFG}	57.922 ± 0.4^{H}	62.1824 ± 0.2^{F}	62.554 ± 1.6^{F}	50.781 ± 0.8^{H}
NARITA23	63.681 ± 0.6^{FGH}	58.909 ± 0.6^{G}	61.946 ± 0.6^{F}	57.52 ± 2.0^{GH}	59.46 ± 1.2^{EF}
Mbiide	63.561 ± 0.8^{GH}	59.136 ± 0.6^{G}	61.565 ± 0.3^{F}	62.411 ± 1.6^{F}	51.782 ± 0.5^{H}
NAROBAN5[M30]	63.073 ± 0.6^{GH}	58.212 ± 0.5^{H}	61.307 ± 0.4^{F}	62.99 ± 1.9^{EF}	56.473 ± 0.4^{G}
FHIA17	60.49 ± 0.7^{HI}	57.326 ± 0.6^{HI}	68.707 ± 0.3^{D}	83.502 ± 1.3^{C}	90.159 ± 0.8^{C}
Mpologoma	58.506 ± 0.9^{IJ}	55.869 ± 0.3^{HI}	58.144 ± 0.4^{G}	56.42 ± 2.3^{H}	46.937 ± 0.4^{I}
KABANA6H[M9]	55.558 ± 1.3^{J}	51.762 ± 0.5^{JK}	53.233 ± 0.5^{I}	57.118 ± 1.5^{H}	46.849 ± 0.7^{I}
NARITA21	50.604 ± 0.6^{K}	47.808 ± 0.2^{K}	48.912 ± 1.2^{J}	46.888 ± 0.9^{I}	46.72 ± 0.3^{I}

Values are presented as mean values ± standard deviation. Values followed by different letters in the same column differ significantly according to Tukey's test ($p < 0.05$).

Table S2: Intensity ratio (IR) of the crystalline peaks

	IR ratio	
**Sample**	**1045/1022**	**995/1022**
Nfuuka	0.62	1.04
Mbidde	0.68	1.01
Nakitembe	0.70	1.07
Kibuzi	0.73	1.03
Mpologoma	0.74	1.02
KABANA6H	0.69	1.06
NARITA23	0.70	0.95
NARITA21	0.70	1.00
FHIA17	0.72	1.03
NAROBAN5	0.75	1.04
Gros Michel	0.71	1.01
Sukali Ndiizi	0.75	1.01
Gonja	0.69	0.99
Kayinja	0.74	1.09
Kivuvu	0.81	1.07

Table S3: Pasting properties of banana RS

Sample	Peak 1 (cp)	Trough 1 (cp)	Breakdown (cp)	Final Viscosity(cp)	Setback(cp)	Peak Time(Min)	Pasting Temperature(°C)
FHIA17	1661 ± 0^{A}	750 ± 0^{A}	1621 ± 0^{A}	1490 ± 0^{A}	1450 ± 0^{A}	2.67 ± 0^{B}	71.5 ± 0^{DE}
Nfuuka	1577.5 ± 19.1^{B}	411 ± 35.4^{BC}	1521.5 ± 16.3^{AB}	1364.5 ± 116.7^{ABC}	1308.5 ± 81.3^{ABC}	2.77 ± 0.1^{AB}	73.83 ± 1.0^{BCD}
Mpologoma	1534.5 ± 50.2^{B}	425 ± 5.7^{BC}	1464.5 ± 44.5^{BC}	1390 ± 50.9^{ABC}	1320 ± 45.3^{ABC}	2.70 ± 0.1^{AB}	75.63 ± 0.5^{AB}
G. Michel	1516.5 ± 3.54^{B}	458 ± 1.4^{B}	1413.5 ± 2.1^{BCD}	1374 ± 50.9^{ABC}	1271 ± 0^{ABC}	2.70 ± 0^{AB}	72.95 ± 1.1^{CDE}
Gonja	1379 ± 0^{C}	401 ± 0^{BC}	1333 ± 0^{DEF}	1200 ± 0^{BC}	1154 ± 0^{C}	2.60 ± 0^{B}	71.25 ± 0^{E}
Kivuvu	1357 ± 5.66^{C}	350 ± 39.6^{BC}	1362 ± 33.9^{CDE}	1127.5 ± 17.7^{C}	1132.5 ± 21.9^{C}	2.60 ± 0.1^{B}	72.45 ± 0.4^{DE}
Kibuzi	1355 ± 0^{C}	421 ± 0^{BC}	1289 ± 0^{EFG}	1274 ± 0^{ABC}	1208 ± 0^{BC}	2.8 ± 0^{AB}	75.25 ± 0^{ABC}
NARITA21	1306 ± 0^{CD}	395 ± 0^{BC}	1266 ± 0^{EFG}	1135 ± 0^{C}	1095 ± 0^{C}	2.67 ± 0^{B}	71.5 ± 0^{DE}
Kayinja	1272 ± 32.5^{D}	403.5 ± 41.7^{BC}	1223.5 ± 9.2^{FG}	1461.5 ± 23.3^{AB}	1413 ± 18.4^{AB}	2.9 ± 0.1^{AB}	73.93 ± 0.5^{BCD}
Mbiide	1258 ± 0^{D}	374 ± 0^{BC}	1239 ± 0^{FG}	1196 ± 0^{BC}	1177 ± 0^{C}	2.7 ± 0^{AB}	77.25 ± 0^{A}
Nakitembe	1245 ± 0^{DE}	385 ± 0^{BC}	1215 ± 0^{G}	1194 ± 0^{BC}	1164 ± 0^{C}	2.7 ± 0^{AB}	76 ± 0^{AB}
Sukali Ndiizi	1173 ± 15.6^{E}	451.5 ± 77.1^{B}	1076.5 ± 92.6^{H}	1255.5 ± 13.4^{ABC}	1159 ± 90.5^{C}	3 ± 0^{AB}	72.375± 0.5^{DE}
NARITA23	1013 ± 0^{F}	348 ± 0^{BC}	1020 ± 0^{H}	1250 ± 0^{ABC}	1257 ± 0^{ABC}	3 ± 0^{AB}	75.75 ± 0^{AB}
KABANA6H	962 ± 28.3^{F}	415.5 ± 43.1^{BC}	901.5 ± 14.8^{I}	1301 ± 213^{ABC}	1240 ± 17^{ABC}	4.1 ± 1.4^{A}	75.08 ± 1.7^{ABC}
NAROBAN5	949 ± 0^{F}	320 ± 0^{C}	984 ± 0^{HI}	1125 ± 0^{C}	1160 ± 0^{C}	3.07 ± 0^{AB}	76.45 ± 0^{A}

Values are presented as mean values ± standard deviation. Values that do not share the same letter in the same column differ significantly according to Tukey's test ($p < 0.05$).

Table S4: Swelling power (g of water/g of dry sample) across a range of temperatures

Sample	50 °C	60 °C	70 °C	80 °C	90 °C
M9	2.05±0.1	3.9±0.4	6.4±0.4	14.95±1.0	16.05±0.9
M30	3.55±0.1	6.05±0.5	6.49±0.4	17.02±1.5	17.39±0.4
Narita23	3.25±0.3	5.25±0.3	6.10±0.2	16.51±0.5	14.95±0.1
Narita21	3.57±0.4	4.28±0.3	7.10±0.4	15.25±0.3	15.88±0.0
FHIA 17	2.76±0.1	3.59±0.4	5.04±0.2	15.94±0.1	18.43±0.2
Nakitembe	4.12±0.1	5.28±0.3	6.73±0.2	16.95±0.1	17.43±0.2
Mbiide	3.27±0.1	4.58±0.1	6.23±0.3	16.05±0.1	16.70±0.2
Kibuzi	2.32±0.1	3.18±0.2	5.48±0.1	12.90±0.1	12.70±0.4
Mpologoma	3.5±0.0	4.74±0.8	6.78±0.8	15.95±0.1	17.33±0.3
Nfuuka	3.26±0.2	5.01±1.5	5.29±0.6	14.92±0.1	14.46±0.4
Kayinja	2.61±0.1	4.32±0.3	5.23±0.3	15.89±0.0	15.00±0.0
Kivuvu	2.42±0.1	3.99±0.7	5.31±0.0	13.91±0.4	18.73±1.3
Gonja	3.89±0.0	3.81±0.2	6.70±0.2	14.56±0.3	16.48±0.0
Bogoya	3.13±0.6	5.22±0.3	7.13±0.1	15.10±0.1	15.27±0.1
Ndiizi	2.43±0.2	3.93±0.6	5.28±0.3	14.11±0.1	17.75±0.3
Maize	2.01±0.1	3.63±0.3	4.47±0.1	14.00±0.0	14.12±0.1

Table S5: Solubility (g of water/g of dry sample) across a range of temperatures

Sample	50	60	70	80	90
Narita21	4.67±0.47[a]	4.75±0.28[ab]	5.62±1.65[ab]	8.555±0.62[a]	16.71±0.41[a]
Bogoya	4.225±1.09[ab]	5.46±1.75[a]	6.59±0.13[ab]	7.98±0.06[a]	14.22±2.50[abcd]
M30	3.89±2.35[abc]	4.47±0.11[abc]	4.94±0.07[ab]	11.71±3.23[a]	15.65±0.92[ab]
Narita23	3.15±0.21[abc]	2.975±0.04[bcd]	3.89±0.93[ab]	8.61±0.87[a]	12.15±0.21[cde]
Nfuuka	3.125±0.11[abc]	2.975±0.67[bcd]	3.78±0.17[ab]	8.6±0.85[a]	14.65±0.48[abcd]
Mbiide	3.03±0.75[abc]	3.27±0.26[abcd]	4.73±0.23[ab]	9.73±0.39[a]	12.89±0.16[bcde]
Mpologoma	3.00±0.00[abc]	3.935±0.09[abcd]	5.11±0.79[ab]	7.94±0.08[a]	12.5±0.69[bcde]
FHIA 17	2.975±0.04[abc]	3.125±0.17[bcd]	4.75±1.06[ab]	10.25±0.35[a]	13.45±0.64[abcde]
Gonja	2.575±0.8[abc]	2.475±0.67[bcd]	3.9±0.49[ab]	10.52±1.38[a]	12.73±0.32[bcde]
Kibuzi	2.44±0.17[abc]	3.49±0.69[abcd]	6.76±0.33[a]	8.55±0.78[a]	13.83±0.25[abcde]
M9	2.415±0.16[abc]	3.475±0.67[abcd]	2.22±2.52[b]	7.56±0.46[a]	10.75±0.64[ef]
Kivuvu	2.26±0.09[abc]	2.465±0.05[bcd]	2.71±0.58[ab]	10±2.84[a]	14.95±1.34[abcd]
Kayinja	2.17±0.24[abc]	2.475±0.67[bcd]	4.57±1.58[ab]	10.28±3.22[a]	14.18±0.88[abcde]
Nakitembe	2.15±0.21[abc]	2.925±0.08[bcd]	4.54±0.76[ab]	7.72±0.36[a]	11.62±0.54[de]
Ndiizi	1.98±0.04[abc]	2.125±0.18[d]	3.75±1.06[ab]	9.13±0.53[a]	12.9±0.00[bcde]
RS Control	1.56±0.01[bc]	2.285±0.38[cd]	4.85±1.97[ab]	6.48±0.11[a]	8.05±0.56[f]
Maize	1.265±0.05[c]	3.425±0.11[abcd]	5.3±0.42[ab]	7.67±0.31[a]	15.33±0.46[abc]

Values are presented as mean values ± standard deviation. Values followed by different letters in the same column differ significantly according to Tukey's test ($p < 0.05$).

FTIR spectra showing the peak positions in the range of 4500-500cm-1

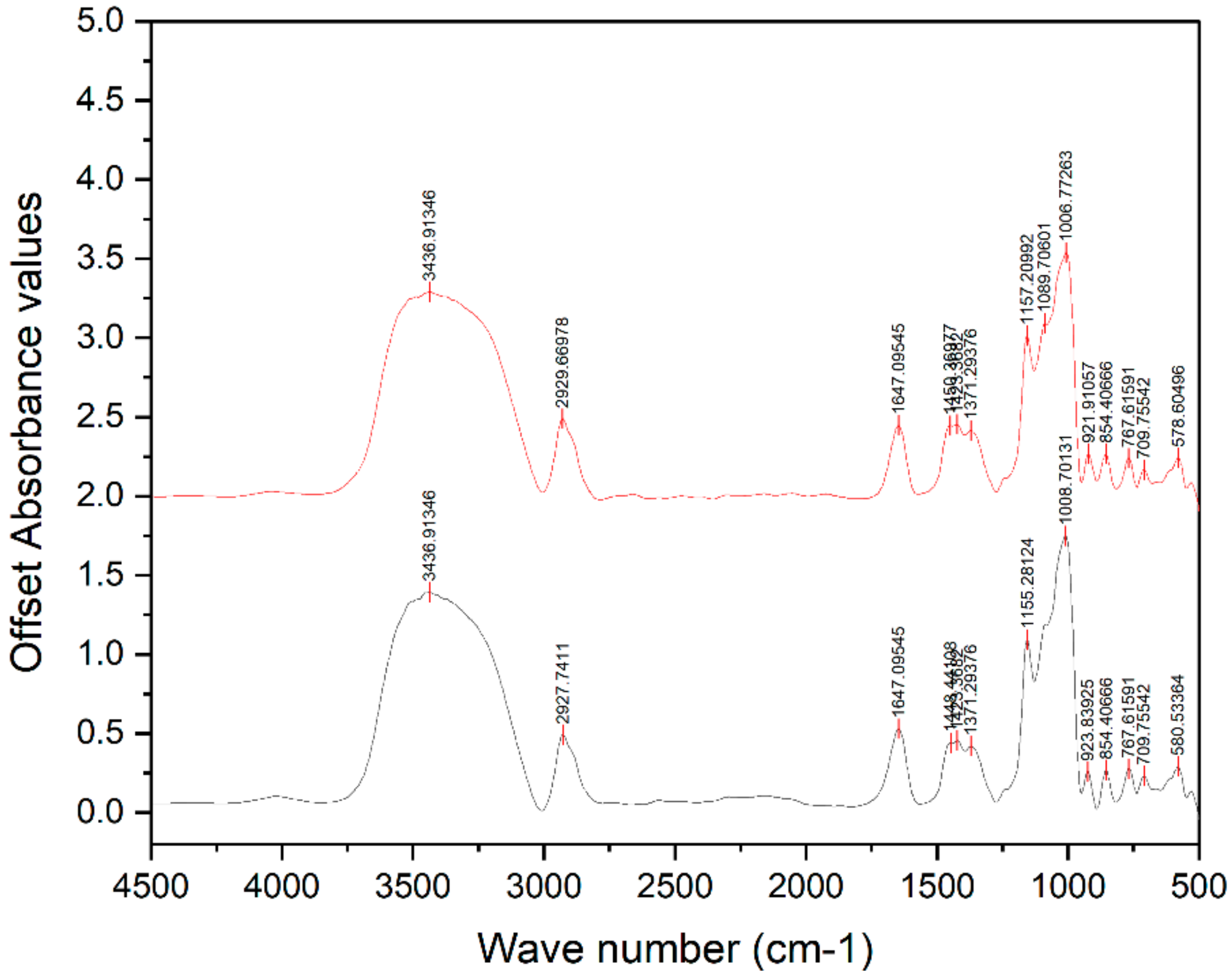

Figure S1: FTIR spectra for RS from the dessert bananas

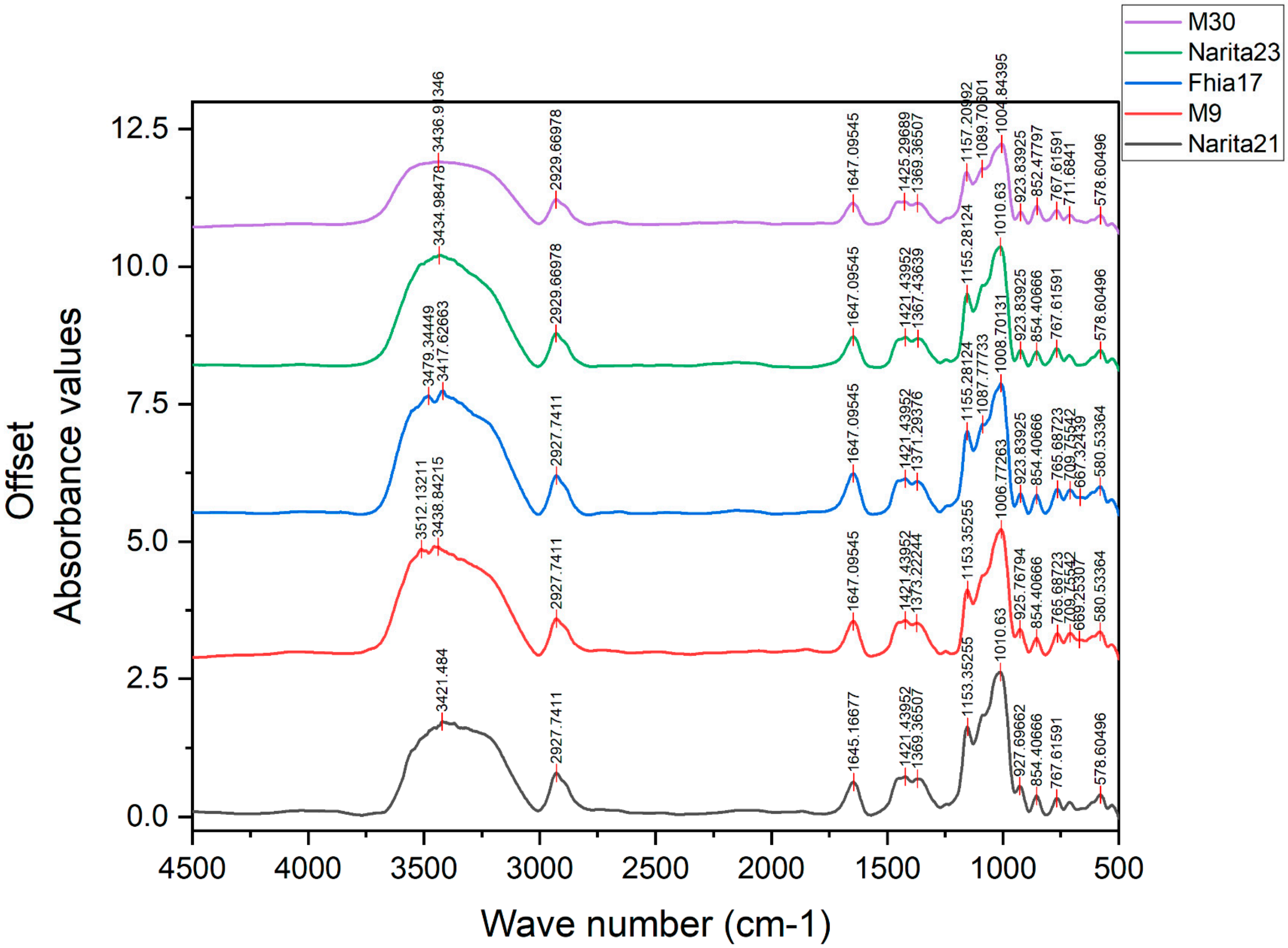

Figure S2. FTIR spectra for the RS for Hybrid bananas

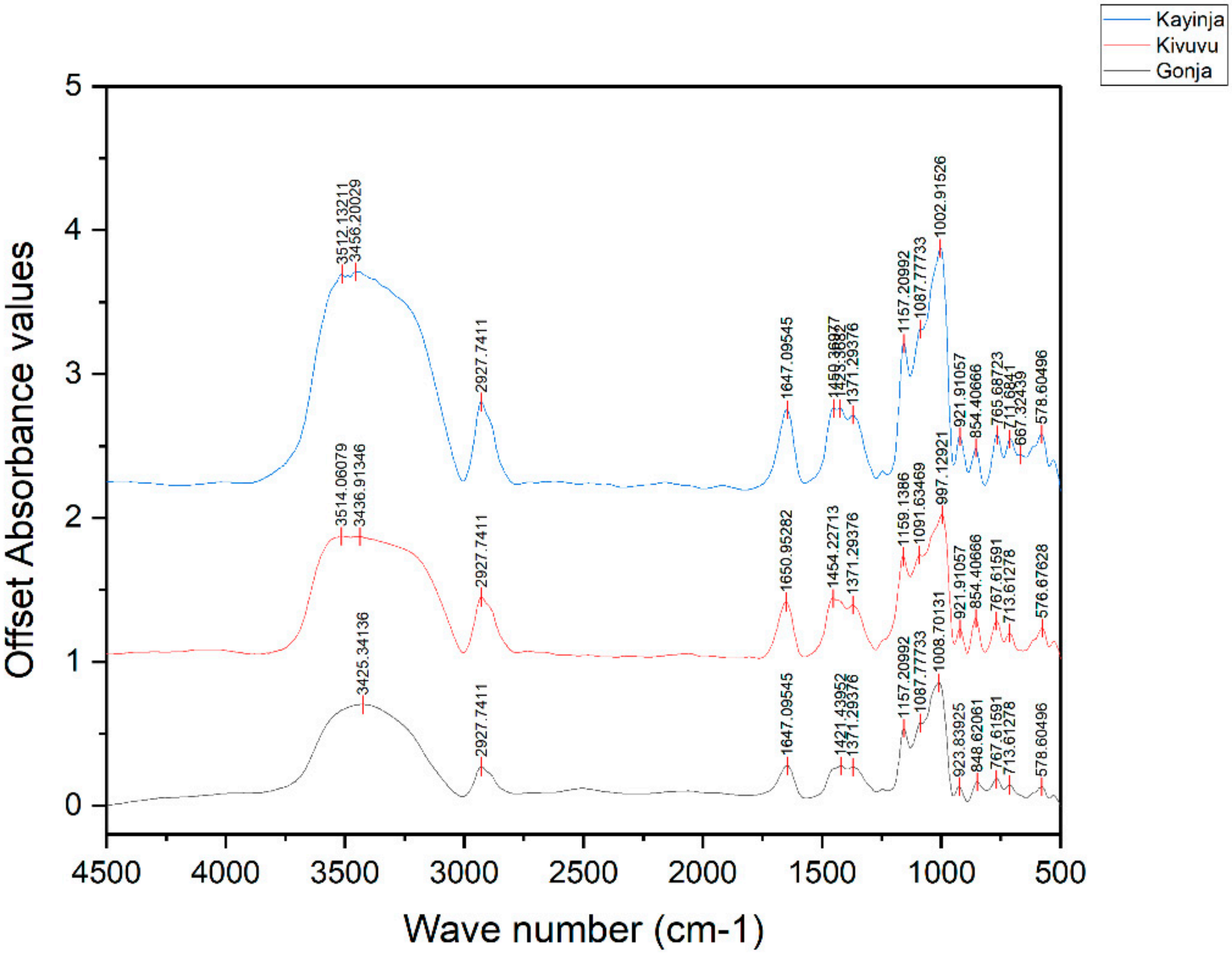

Figure S3. FTIR spectra for the RS from Plantains

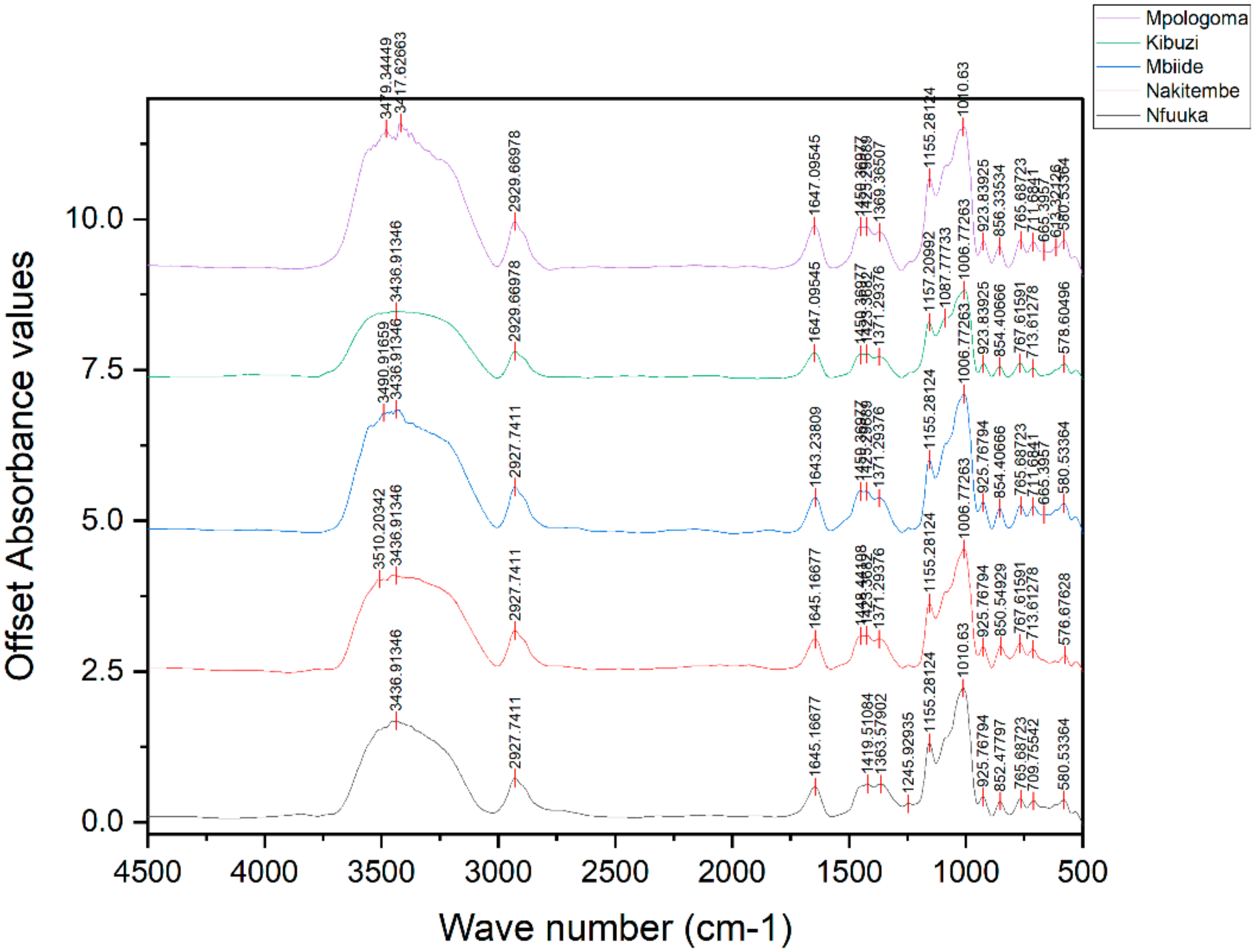

Figure S4.FTIR spectra of the RS from EAHB

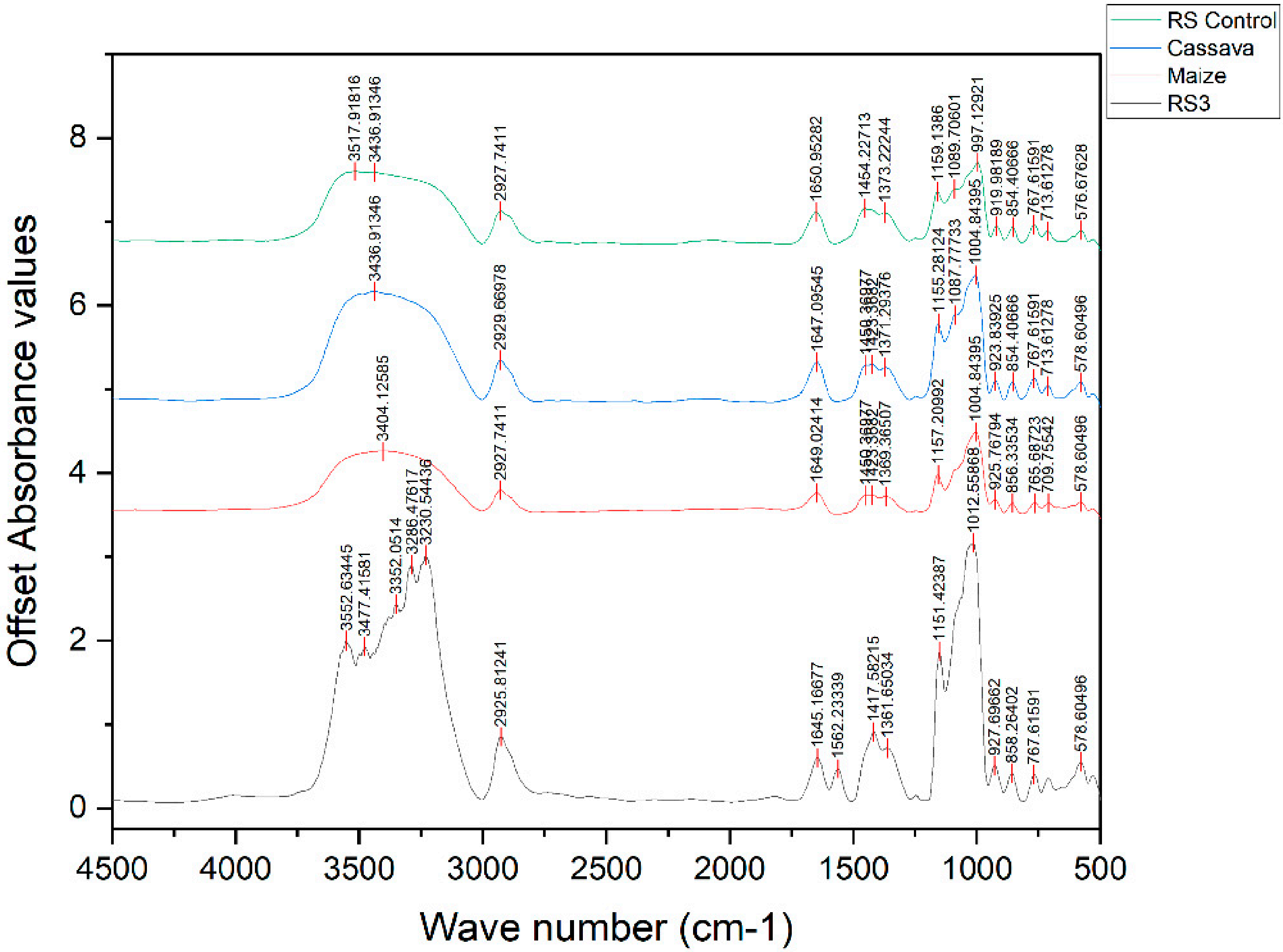

Figure S5. FTIR spectra for the controls

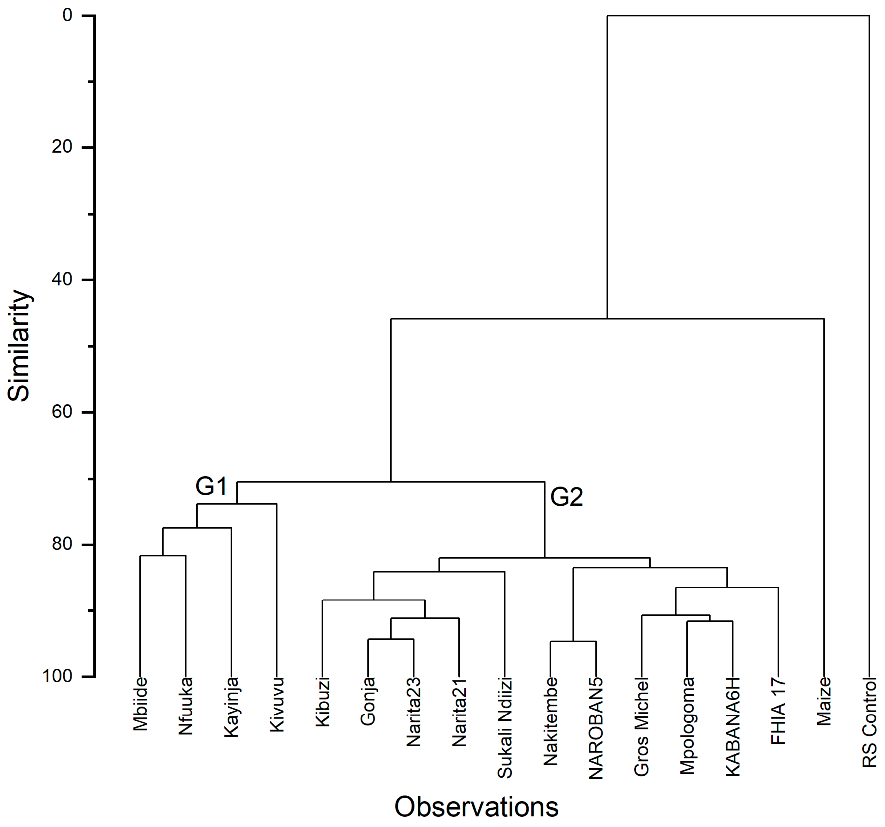

Figure S6: Cluster analysis of the banana cultivars according to polymer composition, RS content and functional properties. Maize is pure, unmodified maize starch while RS control is chemically modified maize starch from Megazyme.

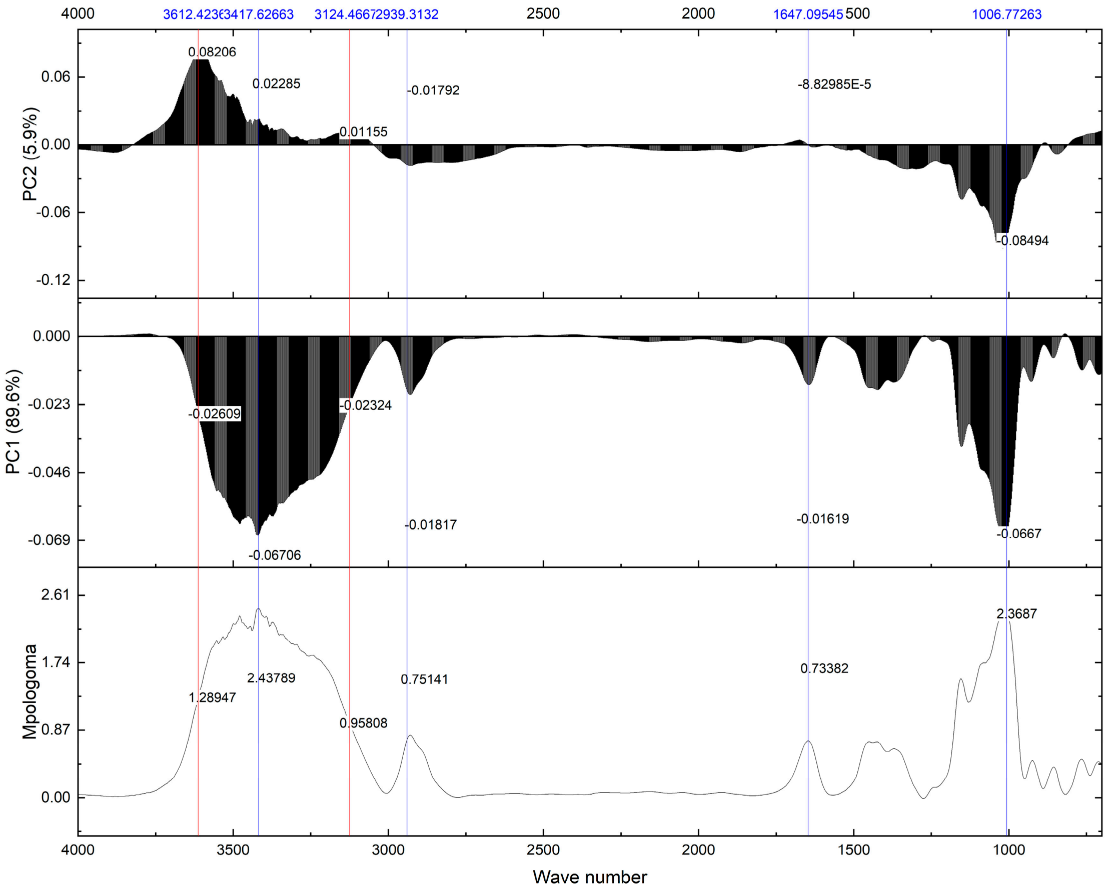

Figure S7: Representative Loading plot highlighting regions used for PCA
